# REPLY BY THE AUTHORS: RE: Parasacral transcutaneous electrical nerve stimulation in children with overactive bladder: comparison between sessions administered two and three times weekly

**DOI:** 10.1590/S1677-5538.IBJU.2021.0341.1

**Published:** 2021-06-03

**Authors:** Maria Luiza Veiga, Kaíse Oliveira, Vanessa Batista, Ananda Nacif, Ana Aparecida Martinelli Braga, Ubirajara Barroso

**Affiliations:** 1 Escola Bahiana de Medicina Departamento de Fisioterapia SalvadorBA Brasil Departamento de Fisioterapia, Escola Bahiana de Medicina, Salvador, BA, Brasil.; 2 Escola Bahiana de Medicina e Saude Publica SalvadorBA Brasil Escola Bahiana de Medicina e Saude Publica Salvador, BA, Brasil.; 3 Universidade Federal da Bahia Departamento de Urologia SalvadorBA Brasil Departamento de Urologia, Universidade Federal da Bahia – UFBA, Salvador, BA, Brasil.

*To the editor,*

We chose not to have a placebo group, as we and others have already demonstrated TENS is effective for OAB in children (1-3). In our article, the main objective was to compare if the application of TENS 2 times a week could be as effective as TENS 3 times a week, since the frequency of TENS sessions is currently empirical (4). As in all the articles published by our group that involved TENS, the children received urotherapy. Therefore, the difference in outcome found between the groups concerns the action of TENS.

Regarding the article on TENS 2 versus 3 times a week, although the DVSS improved after treatment in both groups, there was no difference in the results of the inter-group evaluation. However, as pointed out in the letter to the Editor (5), voiding frequency improved in the bladder diary only in the TENS 2 times a week group. The interpretation, in this case, should not be that twice a week is better than 3 times, which does not make sense. But yes, it draws attention that data from the diary may not be reliable due to the measurement bias and that there may have been a spurious association. There were no intergroup differences in relation to the diary.

Evaluating the effect size for each group separately, we verified a moderate effect size, demonstrating once again that the response was similar between them (TENS 3x/w: Cohen's d- 1.83, size of effect: 0.87; TENS 2x/w: Cohen's d-1.73, size of effect- 0.65).

The Authors
